# High Prevalence of Respiratory Muscle Weakness in Hospitalized Acute Heart Failure Elderly Patients

**DOI:** 10.1371/journal.pone.0118218

**Published:** 2015-02-11

**Authors:** Pedro Verissimo, Karina T. Timenetsky, Thaisa Juliana André Casalaspo, Louise Helena Rodrigues Gonçalves, Angela Shu Yun Yang, Raquel Caserta Eid

**Affiliations:** Intensive Care Unit and Coronary Care Unit, Hospital Israelita Albert Einstein, São Paulo, Brazil; Inserm, FRANCE

## Abstract

**Introduction:**

Respiratory Muscle Weakness (RMW) has been defined when the maximum inspiratory pressure (MIP) is lower than 70% of the predictive value. The prevalence of RMW in chronic heart failure patients is 30 to 50%. So far there are no studies on the prevalence of RMW in acute heart failure (AHF) patients.

**Objectives:**

Evaluate the prevalence of RMW in patients admitted because of AHF and the condition of respiratory muscle strength on discharge from the hospital.

**Methods:**

Sixty-three patients had their MIP measured on two occasions: at the beginning of the hospital stay, after they had reached respiratory, hemodynamic and clinical stability and before discharge from the hospital. The apparatus and technique to measure MIP were adapted because of age-related limitations of the patients. Data on cardiac ejection fraction, ECG, brain natriuretic peptide (BNP) levels and on the use of noninvasive ventilation (NIV) were collected.

**Results:**

The mean age of the 63 patients under study was 75 years. On admission the mean ejection fraction was 33% (95% CI: 31–35) and the BNP hormone median value was 726.5 pg/ml (range: 217 to 2283 pg/ml); 65% of the patients used NIV. The median value of MIP measured after clinical stabilization was -52.7 cmH2O (range: -20 to -120 cmH_2_O); 76% of the patients had MIP values below 70% of the predictive value. On discharge, after a median hospital stay of 11 days, the median MIP was -53.5 cmH_2_O (range:-20 to -150 cmH_2_O); 71% of the patients maintained their MIP values below 70% of the predictive value. The differences found were not statistically significant.

**Conclusion:**

Elderly patients admitted with AHF may present a high prevalence of RMW on admission; this condition may be maintained at similar levels on discharge in a large percentage of these patients, even after clinical stabilization of the heart condition.

## Introduction

Heart failure (HF) is a syndrome that impairs functioning of different organ systems [1]. Patients with HF have central and peripheral factors which affect their exercise capacity [2]. Peripheral factors originating in the vascular, respiratory or muscular systems may decrease the exercise capacity [2]. Myopathy is one of the most common clinical manifestations in these patients. The muscle is subject to structural and functional alterations due to reduction in irrigation by the capillary vessels, decrease of mitochondrial oxidative capacity and increase in angiotensin II levels. In consequence there is atrophy of muscle fibers that leads to substitution of type I muscle fibers by type II fibers; these processes determine progressive reduction in the patients’ exercise capacity due to early fatigue and hyperventilation. The respiratory muscles may also suffer many of these functional alterations [2].

Individuals with HF can develop both peripheral and respiratory muscle weakness [1]. Indeed, several studies have shown alterations in structure and function of respiratory muscles in chronic heart failure (CHF). They showed that the left ventricular dysfunction decreases the ability of CHF patients to exercise and leads to increase in dyspnea during the course of their daily activities. Moreover, these patients often present reduced respiratory muscle strength and endurance. Therefore respiratory muscle abnormalities may worse exertional dyspnea and contribute to early fatigue in patients with CHF [2–5].

AHF patients, when they are hospitalized, may already present variable degrees of muscular impairment and thus present a higher risk of muscle dysfunction as the acute phase of the disease itself requires an obligatory initial bed rest period due to dyspnea [6]. This limitation in mobility is a known and important factor of muscular strength loss [6]. In addition, other factors such as sepsis-induced vascular and metabolic disturbances, malnutrition, neuropathy, myopathy, pharmacological doses of corticosteroids, as well as prolonged inactivity, contribute to the weakening of the skeletal muscles during critical illness. Among these factors, bed rest appears as an important factor of muscle strength loss, which also involves impairment of respiratory muscles [6].

The measurement of maximum inspiratory pressure (MIP) objectively evaluates respiratory muscle strength and has important clinical significance, since MIP has a direct prognostic correlation with mortality in heart failure [7]. Furthermore, it is known that muscle weakness, including respiratory muscle weakness, affects intensive care unit patients [8–10] leading to reduction of functional status and of life quality [11]. However, most studies report on critically ill patients submitted to mechanical ventilation. So far, there are no data available on the occurrence of muscle weakness and/or respiratory muscle weakness in Acute Heart Failure (AHF) patients, especially in those who were not mechanically ventilated.

Inspiratory muscle weakness has a prevalence of 30–50% in outpatients with CHF [4,5]. However, no studies to date have investigated the prevalence of inspiratory muscle weakness in AHF patients. We undertook the present study to investigate the state of respiratory muscle strength in AHF patients admitted to the intensive care unit (ICU) and coronary care unit. The measurement of MIP was used as a parameter to detect inspiratory muscle weakness in these patients. We also investigated whether the clinical improvement resulting from the treatment of AHF and the standard physiotherapy treatment provided during hospitalization would be accompanied by amelioration of the patients’ respiratory muscle strength. To this end, we tested MIP at the discharge from the hospital and compared the data to those on admittance after the patients’ clinical stabilization.

The knowledge regarding the prevalence and the level of inspiratory muscle weakness in critically ill AHF patients is essential, because specific physiotherapeutic and/or medical interventions can be implemented in order to improve and accelerate their recovery. Indeed, the impact of muscle weakness, including inspiratory muscle weakness in critically ill patients in ICU has been the subject of much discussion [9]. To our knowledge, our present study is the first that addresses this issue in hospitalized AHF patients.

## Materials and Methods

### Patients

Patients admitted with AHF were recruited in an ICU and Coronary Care Unit (CCU) at the Hospital Israelita Albert Einstein (HIAE), a major tertiary private hospital in São Paulo-Brazil, and followed up until hospital discharge. The study was conducted in the period of November 2009 to March 2011. Patients included in the study participated in a daily standard physical therapy program.

This study was approved by the HIAE Ethical Committee. Informed consent forms from the patients were waived by the review board, because they were already involved in the HIAE´s heart failure managed protocol, in which measurement of MIP was required.

Inclusion criteria were: to be diagnosed with AHF according to HIAE´s HF management protocols, to present ejection fraction < 45% on echocardiography, to be older than 18 years and to have had standard physiotherapy prescribed during the hospital stay. The HIAE´s HF management protocol for the primary clinical diagnosis follows the Boston [12] criteria and includes one of the following presentations: 1) Acute HF (without a previous diagnosis); 2) Decompensated chronic HF (patients previously diagnosed with HF); 3) Cardiogenic shock; 4) Acute pulmonary edema. All AHF patients were submitted at hospital admission as part of the HIAE standard protocol of AHF evaluation to echocardiogram, determination of plastic levels of brain natriuretic peptide hormone (BNP); body mass index (BMI) were also determined.

Patients presenting any of the following conditions were excluded from the study group: 1) hemodynamic instability and arrhythmias; 2) presence of myocardial necrosis markers and presence of acute ischemic alterations on the electrocardiogram (such as ST-segment elevation or depression); 3) motor abnormalities that compromise locomotion; 4) diagnosis of neurological and neuromuscular conditions (previously diagnosed or diagnosed during hospital stay by neurologists); 5) level of consciousness altered enough to compromise respiratory muscle strength measurements; 6) patients who underwent surgery at hospitalization time; 7) chronic pulmonary diseases; 8) patients intubated or tracheostomized; 9) patients participating in other clinical research protocols; 10) previously reported critically ill condition or muscle weakness.

### Respiratory muscle strength

Respiratory muscle strength was assessed by measuring maximum inspiratory pressure (MIP) using an analogic manuvacuometer (Comercial Medica) attached to an unidirectional valve (Hudson RCI) connected to the face mask (Hudson RCI), with the patients at sitting position [13]. Pressures were maintained for 2 seconds and the plateau level was recorded. The test was repeated three times with 1-min intervals between each one, and the highest value obtained was used for analysis unless it happened to be the last one, in which case a fourth measurement was carried out. The measurement must to be performed from residual volume. The participating physiotherapists were trained to perform MIP determinations using the same apparatus, technique and protocol in order to minimize variation in MIP determinations.

We chose measurement of MIP as the best way to assess respiratory muscle strength in our study, based on previous studies that have used this same parameter to evaluate the effect of respiratory muscle training in heart failure patients [14]. In addition, MIP was also validated as a parameter in a study to determine the reference values of respiratory muscle strength for the Brazilian population [15].

The choice of a mask [13] for the measurement of MIP was decided after observing that many elderly patients were not able to tightly enclose the original mouthpiece with the lips.

The unidirectional valve was used to overcome any difficulty of collaboration and to optimize the voluntary effort of the patient, improving the effectiveness and reproducibility of the MIP test [16,17]. With the use of the unidirectional valve, the patient was required to perform successive efforts from respiratory volumes gradually nearing the residual volume, and would thus generate more negative pressure [16,17].

We evaluated MIP at two time-points. The first was just after the clinical stabilization when the patient was recruited to the study. The clinical stabilization was defined by hemodynamic stability, absence of arrhythmias and also of ischemic ECG alterations (as ST-segment elevation or depression), by the absence of myocardial necrosis markers and by the lack of dyspnea at rest. The second time-point was just before hospital discharge after a variable period of hospitalization.

### Respiratory muscle weakness

The condition of respiratory muscle weakness (RMW) was defined when the MIP was lower than 70% of the predictive value, as described by Dall’Ago et al [3].The value predicted by gender and age was based on the data and calculations reported for the Brazilian population [15].

### Collection from patient records

Demographic data were collected from all the selected subjects from their medical records in the first day of hospitalization and included: age, gender, weight, height and BMI. In addition, we collected echocardiograph data, B-type natriuretic peptide (BNP) levels, and the recorded the use of noninvasive ventilation (NIV).

### Institutional standard physiotherapy protocol

All patients were submitted to a daily program of motor and respiratory physical therapy, twice daily. The exercises consisted of motor exercises for upper limbs including the flexor and extensor muscles of the elbow, flexor and abductor of shoulder. For the lower limbs were included extensor muscles of the knee, extensors, abductors and, adductors of the hip and plantar flexors. Moreover, the patients realized ambulation, up and down stairs. Respiratory exercises consisted of diaphragmatic breathing exercises, intermittent positive pressure breathing and noninvasive ventilation when indicated.

### Statistical analysis

Categorical variables were expressed in absolute and relative frequencies (percentages). Quantitative variables were expressed as the means and their 95% confidence interval (CI 95%) when normally distributed, or as medians and interquartile range (IQR) when conversely distributed. To evaluate the differences between non-parametric variables, we used the Mann Whitney Test to compare MIP values at hospital admission and hospital discharge. The R software, version 10.1 was used for the statistical analysis.

## Results

During the study period 449 patients were admitted due to AHF. Of these patients, 215 (48%) didn’t have physical therapy prescription and were not evaluated; 129 (29%) patients were excluded due to exclusion criteria. We started by evaluating 105 AHF patients. The median MIP measurement at hospital admission was-50 cm H2O (range of-20 to-120). Seventy-three (69.5%) patients had respiratory muscle weakness. Unfortunately only 63 (60%) patients completed the study. Table 1 shows the characteristics of the 63 patients involved in the study. The mean age of the group was 75 years and men (79%) predominated. All patients had an ejection fraction less than 45% (mean 33%) and left ventricular dysfunction. Moreover, the BNP levels obtained were high as well as the frequency of NIV use. Collectively these data indicate that the patients selected for the study were accurately diagnosed with acutely decompensated heart failure according to HIAE’s HF management protocol. The BMI median value was 26.3 suggesting that these patients did not have cachexia. The most prevalent etiology of AHF was ischemic (84%) (Table 1). In addition, standard neurological evaluation did not detect any neurological disorder in this group of patients.

**Table 1:** Characteristics of the 63 patients with acute heart failure.

Variables	
Gender—n (%)	
Female	13 (21)
Male	50 (79)
Age (years)—mean (95% CI)	75 (72–78.8)
Ejection Fraction (%)—mean (95% CI)	33 (31–35)
Use of NIV at admission—n (%)	41 (65)
BNP (pg/ml)—median (IQR)	726.5 (217–2283)
BMI—mean (95% CI)	26.32 (17–30)
AHF etiologies—n (%)	
Amyloidosis	1 (1.6)
Chagasic	1 (1.6)
Valvular	3 (4.8)
Hypertensive	5 (8.0)
Ischemic	53 (84)

BNP:brain natriuretic peptide hormone; NIV:noninvasive ventilation;

CI:confidence of interval; IQR (interquartil range); BMI: Body mass index.

**Fig 1:**
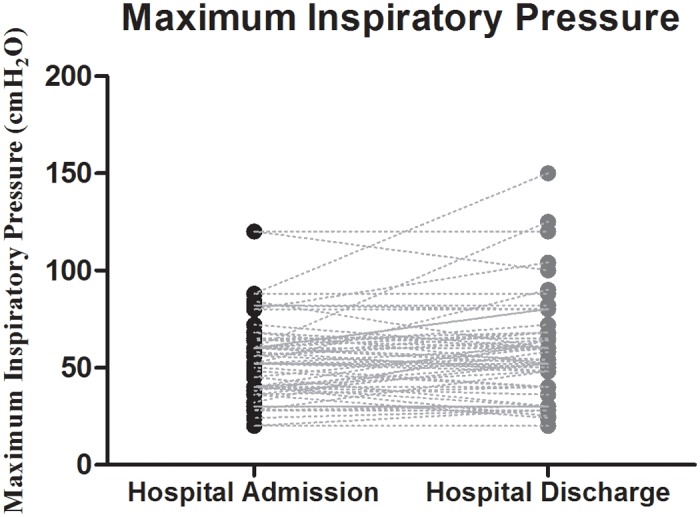
Maximum inspiratory pressure values of the 63 hospitalized acute heart failure patients at hospital admission and hospital discharge.

In order to evaluate the prevalence of respiratory muscle weakness (RMW), we performed MIP measurements on admittance (immediately after the patients clinical stabilization) and on discharge from the hospital. As shown in Table 2, 76% of the patients presented MIP lower than 70% of the predicted value (inferring RMW) at the first time point, and 71% at hospital discharge. Thus, the prevalence of RMW was only slightly modified following recovery of AHF and hospitalization. No significant statistical differences were observed when the MIP values obtained at those time-points were compared (p = 0.806) (Fig. 1). Table 2 also shows that the median time for clinical stabilization was 3.5 days whereas the median hospital length of stay was 11 days.

**Table 2:** MIP measurements and hospital length stay.

Variables	
MIP < 70% of predicted value—n (%)	
Hospital Admission	48 (76)
Hospital Discharge	45 (71)
MIP (cmH_2_O)—median (range)	
First measurement	-53 (-20 to-120)
Hospital discharge	-53 (-20 to-150)
Time to the first MIP measurement (days)—median (IQR)	3.5 (1–22)
Hospital length of stay (days)—median (IQR)	11 (4–36)

MIP:maximum inspiratory pressure; IQR (interquartil range).

## Discussion

Our results in a group of elderly patients with AHF admitted to the ICU and CCU show a high prevalence of inspiratory muscle weakness in these patients. This condition was not modified after recovery from AHF as indicated by the maintenance at the time of hospital discharge of the low levels of MIP observed on admittance.

Inspiratory muscle weakness is an important aspect being discussed regarding the clinical outcome of intensive care patients [18, 19]. Yet, prevalence of inspiratory muscle weakness in AHF is still unknown. The prevalence of inspiratory muscle weakness in our study was high (76%) in patients hospitalized because of AHF. This prevalence was higher than the 50% reported in a previous study that evaluated outpatients with chronic heart failure [3]. However, in our study the patients were older (75yr±11 vs 58yr±2, respectively) and also had lower EF (33%±8 vs 38%±3, respectively) [3]. In another study [20], the reported average prevalence of muscle weakness in CHF patients was 59%. Again the patients were younger (65.7yr±10.5) and had higher mean EF (36.1%±7.6) then ours. These characteristics, older age and lower EF, of our patients may have influenced the higher prevalence of inspiratory muscle weakness. It is important to mention that our patients did not have any neurological disorder that could account for the respiratory weakness.

The acute decompensation of heart disease leads to hypoxemia, systemic inflammation and requires bed rest due to dyspnea and fatigue; these factors may also contribute to a higher prevalence of respiratory muscle weakness in AHF compared to CHF patients [9].

Unexpectedly, our results showed that the percentage of patients with inspiratory muscle weakness still remained high on hospital discharge.

Thus, hospitalization neither improved nor worsened the clinical status of the patients regarding inspiratory muscle weakness. Nevertheless, previous studies have shown that progressive muscle weakness is a common occurrence in critically ill patients [21]. In fact, a muscle biopsy study indicated that the area of skeletal muscle fibers decreases by 2% to 4% per day in critically ill patients admitted to ICUs [10]. A plausible explanation for the maintenance of MIP levels (although low) in our patients may be that the impact of hospitalization was minimized not only by the given medical care but also by the daily physiotherapy program aimed at strengthening the peripheral muscles. It is worth noticing that the group of patients analyzed in this study did not include patients in need of ventilatory support or septic patients, who are the ones who most suffer reduction in muscle strength. It should also be stressed that MIP evaluation in our patients was done using a unidirectional valve [16,17]. This aspect is important because these modifications optimize the voluntary effort of the patient and yield reliable MIP readings [16,17].

On the other hand, it is worth discussing why inspiratory muscle strength did not increase on discharge. Among the factors, the length of hospital stay (median 11 days), the low level of physical activity during the hospitalization due to the patient’s acute disease decompensation, allied to the absence of a specific inspiratory muscle training (IMT) program, may have contributed to the maintenance of the reduced status of inspiratory muscle strength. Indeed, studies on specific IMT outpatients’ programs show that improvement of inspiratory muscle strength and physical capacity were only observed after 8–12 weeks of training [3, 22]. Moreover, it is known that only aerobic training is capable of improving MIP and cardio-respiratory responses to exercise; the addition of specific IMT further enhances this response [23]. Thus, not only the absence of specific IMT, but also the absence of aerobic exercise might have contributed to the non-improvement of respiratory muscle strength in AHF hospitalized patients. It is conceivable that more intense or frequent physical therapy focused on IMT may improve the level of inspiratory muscle strength after clinical stabilization.

Furthermore our results did not detect an association between decreased cardiac function and muscle weakness in our AHF patients, as revealed by the lack of correlation between the EF measurements and MIP corroborating the previously described results by Meyer [7]. So far we have not found additional published studies investigating this correlation. Still, a study that measured the cardiac index in CHF patients, suggests that inspiratory muscle weakness and decreased cardiac function are correlated [24]. However it is possible that EF measurement alone is not sufficient to evidence a correlation between cardiac function and respiratory muscles weakness.

BNP is an important prognostic marker for cardiac patients with AHF [25]. Our study showed high levels of BNP (median: 726.5 pg/ml) in our patients’ group, indicating acutely decompensated heart failure. We did not find a statistically significant correlation between the levels of BNP and respiratory muscle weakness.

Future studies using other parameters to evaluate cardiac function will possibly shed light on this matter.

Use of NIV by the patients in our study was 65%. The common clinical indications for NIV application in AHF are dyspnea, hypoxemia and pulmonary congestion [26]. We did not find a statistically significant correlation between the use of NIV and respiratory muscle weakness. Based on our results, we suggest that this correlation may not exist in our group of patients because patients with AHF that had NIV improved the same respiratory parameters that originally determined NIV but did not increase their MIP levels.

### Study’s limitations

So far the studies on muscle weakness and its consequences in patients with heart failure have reported data from patients on outpatient rehabilitation programs. Therefore the rehabilitation approach that is currently being offered to the inpatient population is mostly based on those results on outpatients. The physiotherapy team involved in the study was trained for the standard protocol treatment and for the respiratory muscle force measurements in order to get less variation in these measurements. Unfortunately we were not able to contact the patients after discharge from the hospital; it would be interesting to have a follow up on MIP and other respiratory parameters. It would also be interesting to have had MIP data before hospitalization, but that was not feasible and beyond the scope of this study. The number of patients in the study was limited because of the difficulty to recruit patients with HF in this age group without the comorbidities considered in the exclusion criteria.

### Clinical impact

This study has an important clinical impact, as it shows a high prevalence of respiratory muscle weakness in hospitalized patients, and stresses the importance of such evaluation, (as this may be a prognostic factor). The measure of MIP has prognostic correlation in HF: patients with lower MIP have worse outcomes [7, 27] The MIP may also be a parameter to indicate the need for respiratory and cardiac rehabilitation after hospital discharge.

It is known that referral to cardiac rehabilitation is a quality parameter recommended by the American College of Cardiology/American Heart Association (ACC / AHA) after hospital discharge [28]; therefore, early detection of respiratory muscle weakness during hospitalization, can facilitate this referral. In addition, some important alterations occur during acute exacerbation of heart failure such as the increased response of the muscle metaboreflex and peripheral chemoreflex, as well as autonomic nervous system dysfunction all of which have been correlated with inspiratory muscle weakness [29–31]. Therefore the measurement of MIP should become even more relevant for inpatients.

A specific inspiratory muscle training program improved the exercise capacity and quality of life of outpatients in rehabilitation programs [3,22,23] This specific inspiratory training may also benefit patients during hospitalization.

Finally, our study indicates that monitoring AHF patients for respiratory condition after hospital discharge seems prudent because they may leave the hospital with inspiratory muscle weakness. Still, more studies are needed to evaluate the impact of specific respiratory training on respiratory muscle strength during the hospitalization period in patients admitted with acutely decompensated heart failure.
