# Correction: High Prevalence of Respiratory Muscle Weakness in Hospitalized Acute Heart Failure Elderly Patients

**DOI:** 10.1371/journal.pone.0124855

**Published:** 2015-04-13

**Authors:** The PLOS ONE Staff

The authors are listed out of order. Please view the correct author order, affiliations, and citation here:

Pedro Verissimo, Thaisa Juliana André Casalaspo, Louise Helena Rodrigues Gonçalves, Angela Shu Yun Yang, Raquel Caserta Eid, Karina T. Timenetsky

Intensive Care Unit and Coronary Care Unit, Hospital Israelita Albert Einstein, São Paulo, Brazil

Verissimo P, Casalaspo TJA, Gonçalves LHR, Yang ASY, Eid RC, Timenetsky KT (2015) High Prevalence of Respiratory Muscle Weakness in Hospitalized Acute Heart Failure Elderly Patients. PLoS ONE 10(2): e0118218. doi: 10.1371/journal.pone.0118218
